# Unraveling the Mechanism of Purple Leaf Formation in *Brassica napus* by Integrated Metabolome and Transcriptome Analyses

**DOI:** 10.3389/fpls.2022.945553

**Published:** 2022-07-12

**Authors:** Haibo Li, Yi Du, Jinkun Zhang, Huimin Feng, Jianguo Liu, Guiling Yang, Yunna Zhu

**Affiliations:** Guangdong Provincial Key Laboratory of Utilization and Conservation of Food and Medicinal Resources in Northern Region, Shaoguan Mustard Engineering Technology Research and Development Center, Henry Fok College of Biology and Agriculture, Shaoguan University, Shaoguan, China

**Keywords:** anthocyanin, purple leaves, *Brassica napus*, metabolome, transcriptome

## Abstract

*Brassica napus* as both oilseed and vegetable, is widely cultivated in China. The purple leaf of *B. napus* is rich in anthocyanins and can provide valuable nutrients. Although several high-anthocyanin cultivars have been reported, the molecular mechanism underlying anthocyanin biosynthesis in *B. napus* remains lesser-known. Therefore, in this study, we conducted integrative metabolome and transcriptome analyses in three *B. napus* cultivars with different leaf colors. Overall, 39 flavonoids were identified (including 35 anthocyanins), and 22 anthocyanins were differentially accumulated in the leaves, contributing to the different leaf colors. Cyanidin-3,5,3’-O-triglucoside was confirmed as the main contributor of the purple leaf phenotype. Meanwhile, other anthocyanins may play important roles in deepening the color of *B. napus* leaves. A total of 5,069 differentially expressed genes (DEGs) and 32 overlapping DEGs were identified by RNA-sequencing; hence, the correlation between anthocyanin content and DEG expression levels was explored. Two structural genes (*DFR* and *ANS*), three *GSTs* (homologous to *TT19*), and 68 differentially expressed transcription factors (TFs), especially MYB-related TFs and WRKY44, were identified in three *B. napus* varieties characterized by different leaf color, thereby indicating that these genes may contribute to anthocyanin biosynthesis, transport, or accumulation in *B. napus* leaves. The findings of study provide important insights that may contribute to gaining a better understanding of the transcriptional regulation of anthocyanin metabolism in *B. napus*.

## Introduction

Anthocyanins are water-soluble pigments that provide color to specific organs, such as flowers, fruits, seeds, and leaves in almost all vascular plants (Landi et al., 2015), thereby conferring diverse colors, including orange, red, violet, and blue (Khoo et al., 2017). In addition to providing beautiful pigmentation to plant organs and attracting pollinators, anthocyanins may protect photosynthetic tissues from oxidative stress induced by UV or visible light (Glover and Martin, 2012). Indeed, plants can induce anthocyanin production under stress conditions, such as wounding, chilling, sulfur limitation, pathogen attack, or nutrient deficiency (Weese et al., 2015). Several studies have revealed that foods rich in anthocyanins provide beneficial health effects in the context of specific disease conditions, such as cancer and cardiovascular diseases, owing to their high antioxidant activity (Khoo et al., 2017; Mattioli et al., 2020).

Anthocyanins are a subclass of flavonoids that contribute to the formation of various color tones. There are six major classes of anthocyanidins, i.e., pelargonidin, cyanidin, peonidin, delphinidin, petunidin, and malvidin (Tanaka and Brugliera, 2013). The biosynthetic pathways of anthocyanins have been well-characterized and are relatively conserved among seed plants (Glover and Martin, 2012). The anthocyanin biosynthetic enzymes belong to various enzyme families, including the first committed enzyme chalcone synthase (CHS), chalcone isomerase (CHI) for flavanones formation, flavanone 3-hydroxylase (F3H) for hydroxylation of the C-ring at the 3-position, flavonoid 3′-hydroxylase (F3′H) and flavonoid 3′,5′-hydroxylase (F3′5′H) which determine the hydroxylation pattern of the B-ring of flavonoids and are necessary for cyanidin and delphinidin production respectively, dihydroflavonol 4-reductase (DFR) for leucoanthocyanidin biosynthesis, and the last one enzyme—anthocyanidin synthase (ANS) for synthesizing the corresponding colored anthocyanidins. Currently, most known anthocyanins are methylated, acylated, or anthocyanidin glycosylated at multiple positions by methyltransferases (MT), acyltransferases (AT), and glucosyltransferases (GT), respectively (Tanaka and Brugliera, 2013). After various modifications, anthocyanins are processed on the cytosolic surface of the endoplasmic reticulum and subsequently sequestered into vacuoles by anthocyanin transporters, such as glutathione S-transferases (GSTs) (Zhao, 2015; Mattioli et al., 2020). Moreover, the final color of anthocyanins depends on vacuolar pH, cell shape, light, and other environmental factors (Enaru et al., 2021).

Anthocyanin biosynthesis is primarily controlled at the transcriptional level. In *Arabidopsis*, the spatial and temporal expression of structural genes involved in anthocyanin biosynthesis is determined by individual R2R3-MYB transcription factors (MYB11, MYB12, and MYB111) or a protein complex comprising R2R3-MYB (production of anthocyanin pigment 1 [PAP1], production of anthocyanin pigment 2 [PAP2], MYB113, and MYB114), basic helix–loop–helix (bHLH) (glabrous, [GL3], enhancer of glabrous [EGL3], and transparent testa 8 [TT8]), and WD40-type (transparent testa glabrous [TTG1]) transcription factors and their interactions (Ramsay and Glover, 2005; Gonzalez et al., 2008). Not all three components of the MYB-bHLH-WD40 (MBW) complex are required for anthocyanin biosynthesis in any species (Katia and Chiara, 2011). For instance, *MYB111*, *TT8*, and one transporter gene (*TT19*) may be responsible for anthocyanin biosynthesis in the high-anthocyanin cultivars *Brassica napus* that is resynthesized by crossing the rich-anthocyanin *Brassica rapa* and *Brassica oleracea* (Goswami et al., 2018). Whole-genome and comparative expression analyses have shown that the upregulation of TT8, along with its target genes, plays an important role in the formation of purple leaf at the early development stage of *B. napus* (He et al., 2021b). However, the regulation of the flavonoid pathway in *B. napus* and the molecular basis for the different leaf colors of *B. napus* are not fully understood.

Herein, we detected and quantified the composition and content of anthocyanins in three different colored leaves of *B. napus* while also elucidating the regulatory network underlying anthocyanin biosynthesis in *B. napus* using an integrated metabolome and RNA-sequencing (RNA-seq) strategy. In this study, we identified candidate genes associated with regulating the mechanism of purple leaf formation in *B. napus*, thereby providing a foundation for metabolic engineering of anthocyanin biosynthesis in *B. napus* leaves.

## Materials and Methods

### Plant Materials

The original *B. napus* mutant with purple leaves (ZH) was found in Huazhong Agricultural University. Female ZH were reciprocally crossed with a male green leaf *B. napus* line (Zhongshuang 11, ZS) to produce F_1_ progeny (ZH × ZS) with reddish-green leaves. The [(ZH × ZS) × ZS] BC_1_ population was obtained by backcrossing F_1_ and ZS; the [(ZH × ZS) × ZS] BC_7_F_2_ population was obtained by backcrossing the individuals with reddish-green leaves with ZH for seven times and by self-pollinating. Individuals with reddish-green leaves from the BC_7_F_2_ population were self-pollinated to [(ZH × ZS) × ZS] BC_7_F_2_S_1_, which produced purple leaf type (PLT), reddish-green leaf type (RGLT), and green leaf type (GLT) individuals. The seedlings of PLT, RGLT, and GLT of *B. napus* were planted in a plastic greenhouse at Shaoguan University (24.8° N and 113.7° E, China), in which temperature ranged between 10 and 20°C and the plants were exposed to natural solar radiation. After 45 d of sowing, the seedlings of *B. napus* had reached the four- to five-leaf stage. Amongst these, we collected 20 fully expanded healthy 2nd and 3rd leaves from 10 plants between 09:00 and 11:00, which were subsequently pooled to give a composite sample. For each *B. napus* variety, we assessed three biological replicates. Each composite sample was subsequently divided into three portions for total anthocyanin content, metabolome and RNA-seq analyses, respectively. All samples were immediately frozen in liquid nitrogen and stored at –80°C.

### Measurement of the Total Anthocyanin Content

The total anthocyanin content of three *B. napus* leaves was extracted in a mixture of 95% methanol and 1.5 mol L^–1^ HCl (85:15, v/v) and quantified according to a method described by Li et al. (2016). For each *B. napus* variety, we assessed three biological replicates. Data were analyzed using a *t*-test and *P* < 0.01 was considered significant.

### Metabolite Extraction and Profiling

Sample preparation, extract analysis, metabolite identification and quantification were performed at Wuhan Metware Biotechnology Co., Ltd. (Wuhan, China). The freeze-dried samples (oilseed leaves stored at –80°C) were crushed using a mixer mill with a zirconia head (MM400, Retsch) at 30 Hz for 1.5 min; 50 mg of powder was weighted and extracted with 0.5 mL 70% methanol at 4°C for overnight. The extract was then ultrasonicated for 5 min and centrifugation at 12,000 × *g* under 4°C for 10 min. The supernatants were collected and filtered through a microporous membrane (0.22 μm) before liquid chromatography (LC)-mass spectrometry (MS) analysis, performed with an ultra-performance liquid chromatography system (Shim-pack UFLC Shimadzu CBM30A) and a tandem MS system (Applied Biosystems 6500 Q TRAP), equipped with an ESI Turbo Ion-Spray interface. The conditions of LC-ESI-MS/MS system were as described by Dong et al. (2019) and Yang et al. (2020). Metabolite data analysis was conducted with the Analyst 1.6.3 software (AB Sciex, ON, Canada). For each *B. napus* variety, we assessed three biological replicates.

Metabolites were identified by comparing the mass/charge (m/z) values, retention time, and fragmentation patterns with the standards hosted on the database curated by Metware Biotechnology Co., Ltd. The supervised multivariate method, orthogonal projections to latent structures-discriminant analysis (OPLS-DA), was used to maximize the metabolome differences between the sample pairs. Differentially accumulated metabolites (DAMs) with variable importance in the project (VIP) ≥ 1 and fold change ≥ 2 or ≤ 0.5 were considered as significantly changed metabolites (Jiao et al., 2020; Fu et al., 2021).

### RNA Extraction and RNA-Seq

All leaves were ground on dry ice to extract the total RNA with a TaKaRa MiniBEST Plant RNA Extraction Kit (No. 9769; Takara Bio, Tokyo, Japan). The quality and integrity of the total RNA were then assessed using the RNA Nano 6000 Assay Kit of the Bioanalyzer 2100 system (Agilent Technologies, CA, United States).

Three micrograms RNA per sample were used as input material for the RNA sample preparations. Sequencing libraries were generated using NEBNext^®^ Ultra™ RNA Library Prep Kit for Illumina^®^ (NEB, MA, United States) following manufacturer’s recommendations, and index codes were added to attribute sequences to each sample. Custering of the index-coded samples was performed on a cBot Cluster Generation System using the TruSeq PE Cluster Kit v3-cBot-HS (Illumina, CA, United States), according to the manufacturer’s instructions. After cluster generation, the libraries were sequenced on an Illumina Hiseq™ platform, and 125 bp/150 bp paired-end reads were generated. For each *B. napus* variety, we assessed three biological replicates.

### RNA-Seq Data Analysis and Annotation

Raw data (raw reads) in fastq format were first processed through in-house Perl scripts. In this process, clean data (clean reads) were obtained by removing reads containing adapter, ploy-N and low-quality reads from raw data. The Q20, Q30, and GC contents of the clean data were simultaneously calculated. All downstream analyses were based on high quality clean data. The clean reads were mapped to the reference genome (Chalhoub et al., 2014) using HISAT 2.2.4 (Kim et al., 2015). The mapped reads of the three sample groups were assembled by StringTie v1.3.1, and the fragments per kilobase of transcript per million mapped reads (FPKM) value was evaluated to quantify expression (Pertea et al., 2016). All transcripts were annotated from the Gene Ontology (GO), Kyoto Encyclopedia of Genes and Genomes (KEGG), NCBI non-redundant (Nr), Swiss-Prot, and Pfam databases. KEGG and GO enrichment analyses were performed using Omicshare.^1^ Genes with a false discovery rate (FDR) < 0.05, absolute fold change ≥ 2, and fold change ≤ 0.5 were considered differentially expressed genes (DEGs). DEGs among the three group samples were identified by DESeq2 for subsequent analyses (Love et al., 2014). A heatmap of DAMs or DEGs was constructed using TBtools, in accordance with the protocol described by Chen et al. (2020).

### Validation of RNA-Seq Using Quantitative Real-Time PCR

The cDNA from different samples were synthesized with GoScript™ Reverse Transcription Mix (Promega, Beijing, China). Quantitative real-time (qRT) PCR was performed using SYBR^®^ Premix Ex Taq™ (TaKaRa Bio) on the CFX Connect Real-Time PCR System (Bio-RAD, CA, United States). The primer pair sequences were listed in Supplementary Table 1. The expression of six anthocyanin biosynthetic genes, one anthocyanin transport gene, and two regulatory genes was evaluated using qRT-PCR of RNA-seq samples to validate the results of RNA-seq. *ACTIN7* (*BnaA02g00190D*) and *GAPDH* (*BnaC03g33610D*) were used as internal controls. Relative gene expression was calculated using the 2^–ΔΔCT^ method (Livak and Schmittgen, 2001). All experiments involved three biological replicates.

## Results

### Total Anthocyanin Content in *Brassica napus* Leaves

**FIGURE 1:**
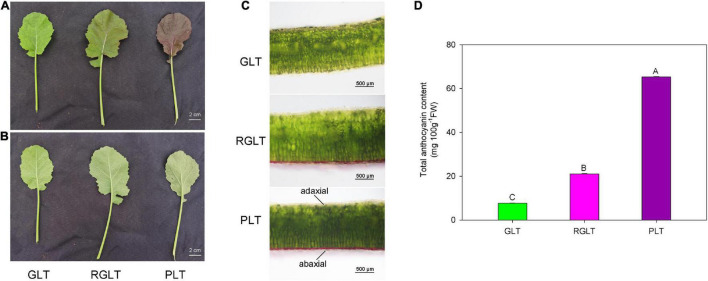
Leaf morphology and total anthocyanin content in three *B. napus* varieties. **(A)** Upper epidermis of green leaf type (GLT), reddish-green leaf type (RGLT), and purple leaf type (PLT). Scale Bars = 2 cm. **(B)** Lower epidermis of GLT, RGLT, and PLT. **(C)** Transverse section of GLT, RGLT, and PLT. Scale Bars = 500 μm. **(D)** Total anthocyanin contents in GLT, RGLT, and PLT. The different capital letters indicate significant differences at *P* ≤ 0.01.

Compared with GLT, both RGLT and PLT exhibited higher levels of anthocyanin accumulation in leaves, whereas that in PLT was higher than that in RGLT (Figures 1A–C). In fact, the total anthocyanin content of PLT was 3.11- and 8.53-fold higher than that of RGLT and GLT, respectively (Figure 1D). Anthocyanin primarily accumulated on the adaxial epidermis of leaves, not on the abaxial side (Figures 1A–C).

### Anthocyanin Metabolites in the Leaves of *Brassica napus*

**FIGURE 2:**
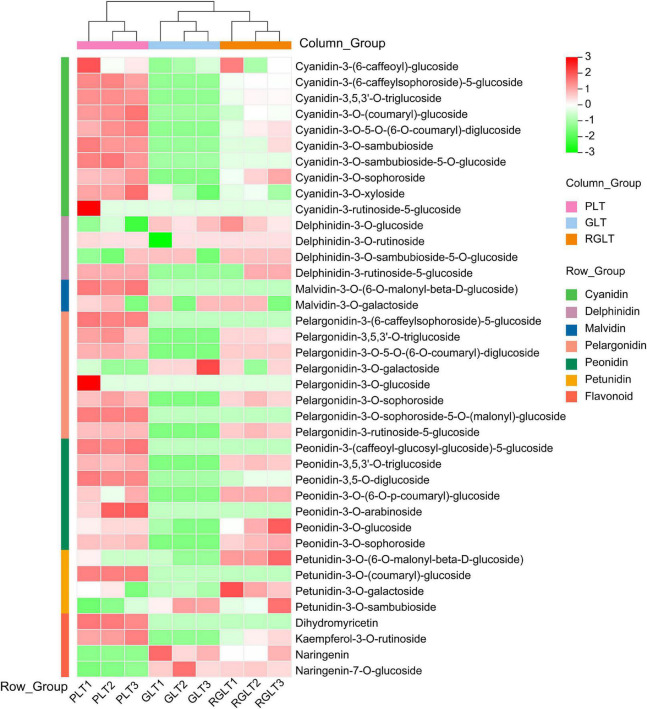
Heatmap of anthocyanin metabolites in the three different colored leaves of *B. napus*. PLT, purple leaf type; GLT, green leaf type; RGLT, reddish-green leaf type. GLT, green leaf type; RGLT, reddish-green leaf type; PLT, purple leaf type.

The data obtained using the API 6500 QTRAP UPLC/MS/MS system were analyzed to compare the anthocyanin metabolites that were differentially expressed among GLT, RGLT, and PLT. A total of 39 different anthocyanin metabolites, including cyanidin, delphinidin, petunidin, peonidin, pelargonidin, and malvidin, were identified in the leaves of three *B. napus* cultivars (Supplementary Table 2). A heatmap of the metabolites was created using R software after unit variance scaling; hierarchical cluster analysis was performed on the accumulation pattern of metabolites among different samples. As shown in Figure 2, the 39 anthocyanin metabolites were classified into seven categories, namely, cyanidins (10), pelargonidins (8), peonidins (7), delphinidins (4), flavonoids (4), petunidins (4), and malvidins (2). The three *B. napus* varieties exhibited different compositions and percentages of anthocyanin metabolites; cyanidins accounted for 31.40–48.68% of the total anthocyanins in RGLT and PLT, and 1.11% in GLT, whereas delphinidins and petunidins accounted for 40.55–53.22% of the total anthocyanins in GLT and 17.66–38.32% in RGLT and PLT (Figure 2 and Supplementary Table 2).

**FIGURE 3:**
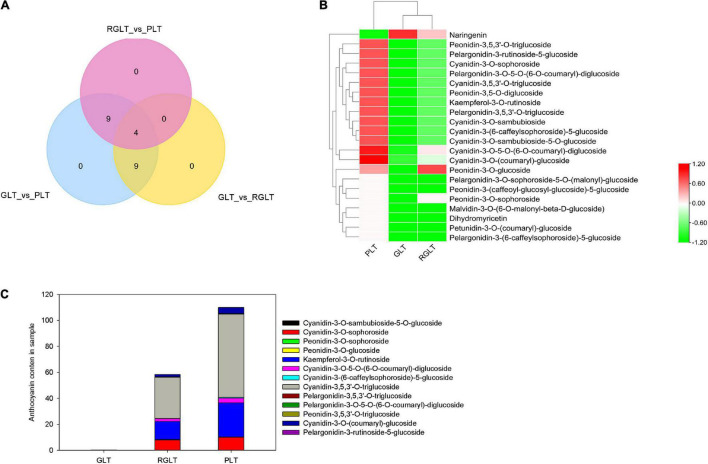
Metabolome analysis of differentially accumulated metabolites (DAMs). **(A)** Venn diagram analysis among GLT vs. PLT, GLT vs. RGLT, and RGLT vs. PLT. **(B)** Cluster heat map of DAMs among GLT, RGLT, and PLT. **(C)** The relative content of overlapping DAMs (four anthocyanins) in the leaves of the three *B. napus* cultivars.

Among these metabolites, 22 DAMs exhibited significant differences in the leaves of the three *B. napus* cultivars. Venn diagram analysis showed that GLT vs. PLT shared nine DAMs with GLT vs. RGLT and nine with RGLT vs. PLT. Moreover, four DAMs were shared among GLT vs. PLT, GLT vs. RGLT, and RGLT vs. PLT (Figure 3A). The cluster heatmap of the DAMs were presented shown in Figure 3B. Except for naringenin, the remaining 21 monomeric anthocyanins in PLT were significantly more abundant than that in GLT, especially cyanidin-3-O-sophoroside, kaempferol-3-O-rutinoside, cyanidin-3-O-5-O-(6-O-coumaryl)-diglucoside, cyanidin-3-(6-caffeylsophoroside)-5-glucoside, cyanidin-3,5,3’-O-triglucoside, and cyanidin-3-O-(coumaryl)-glucoside (Log_2_FC > 5.0; Supplementary Figure 1A). Similarly, the levels of naringenin were lower and those of other monomeric anthocyanins were higher in RGLT than in GLT (Figure 3B and Supplementary Figure 1B). The relative content of overlapping DAMs between GLT vs. PLT and GLT vs. RGLT was detailed in Figure 3C. Anthocyanin classification results showed that most DAMs were cyanidin glucosides, in particular cyanidin-3,5,3’-O-triglucoside, whereas others were peonidin or pelargonidin glucosides (Figure 3B). Hence, the content and composition of these monomeric anthocyanins likely contribute to the differences in *B. napus* leaf color.

### RNA-Seq and Mapping of Transcripts

To analyze genome-wide gene expression and the changes responsible for pigment formation and accumulation we performed RNA-seq analysis on GLT, RGLT, and PLT. After removing low-quality reads, 46,556,414–47,880,324 clean reads were obtained. The percentages of Q20 and Q30 values of each library were 96.86–97.21% and 91.65–92.51%, respectively; the GC content of each sample ranged from 46.49 to 47.69% (Supplementary Table 3), indicating that the quality of RNA-seq data was high. To identify genes corresponding to the reads in each library, the clean reads were mapped to the *B. napus* cv. Darmor-*bzh* reference genome (Chalhoub et al., 2014) using HISAT2.2.4. A total of 91.68–92.47% of the clean reads matched to either a unique or multiple genomic positions, and more than 86.19% uniquely matched reads were used for gene expression analysis of each library (Supplementary Table 4). Most of the uniquely mapped reads were distributed in the exon region (approximately 95.47–96.39%) and intergenic region (2.90–3.79%) with a few in the intron region (0.67–0.90%; Supplementary Table 5). Finally, the sequence and expression information of 101,040 genes was obtained for subsequent analysis.

### Screening of Differentially Expressed Genes

**FIGURE 4:**
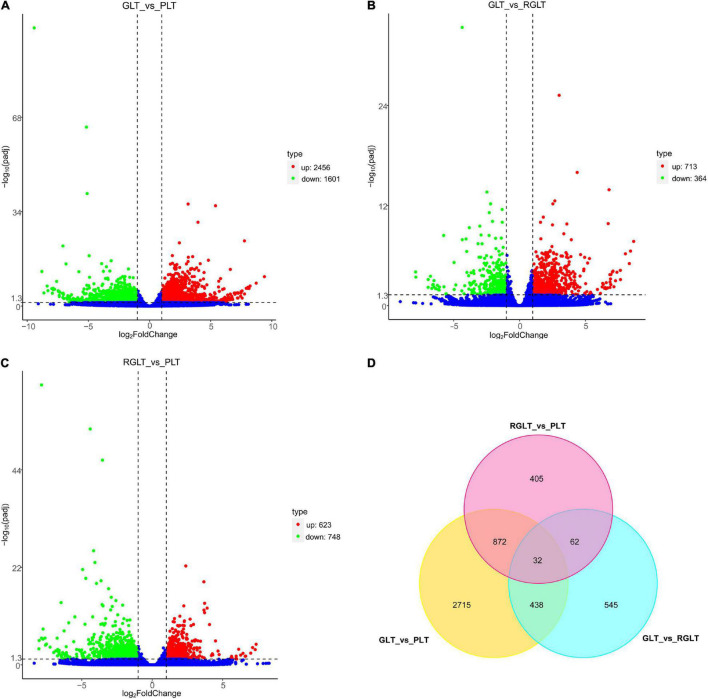
Volcano map and Venn diagram analysis of differentially expressed genes (DEGs) in the different colored leaves of *B. napus*. Red dots indicate upregulated DEGs, green dots indicate downregulated DEGs, and blue dots indicate genes that were not differentially expressed. **(A–C)** Volcano map of DEGs among GLT vs. PLT, GLT vs. RGLT, and RGLT vs. PLT. **(D)** Venn diagram analysis of DEGs among GLT vs. PLT, GLT vs. RGLT, and RGLT vs. PLT.

The correlation between the sample groups and biological replicates was closer to 1.00; thus, Supplementary Figure 2A directly reflected the significant difference between the three groups, indicating clear groupings. DEGs were analyzed using DESeq2 (|Log_2_FC| ≥ 2 and FDR ≤ 0.05), and 5,069 genes were identified as differentially expressed. Compared with the GLT group, 2456 and 713 DEGs were upregulated, 1,601 and 364 DEGs were downregulated in the PLT and RGLT groups, respectively; whereas 713 and 364 DEGs with upregulated and downregulated in the PLT group in comparison to the RGLT group (Figures 4A–C and Supplementary Figure 2B). Furthermore, compared with the RGLT group, 623 DEGs were upregulated and 748 DEGs were downregulated in PLT group (Figure 4C and Supplementary Figure 2B). The results showed that the number of DEGs was the highest in the GLT vs. PLT group, whereas the number of DEGs in the RGLT vs. PLT group was higher than that in the GLT and RGLT group (Figures 4A–C).

In detail, there were 4,057 DEGs between the PLT and GLT groups, 1077 DEGs between the RGLT and GLT groups, and 1,371 DEGs between the RGLT and PLT groups, comprising 904 overlapping genes (872 DEGs between GLT vs. PLT and RGLT vs. PLT groups, and 32 DEGs among GLT vs. PLT, GLT vs. RGLT, and RGLT vs. PLT groups; Figure 4D). Hence, the formation of different colored leaves of *B. napus* might be regulated by these DEGs.

### Functional Annotation and Expression Patterns of Differentially Expressed Genes

**FIGURE 5:**
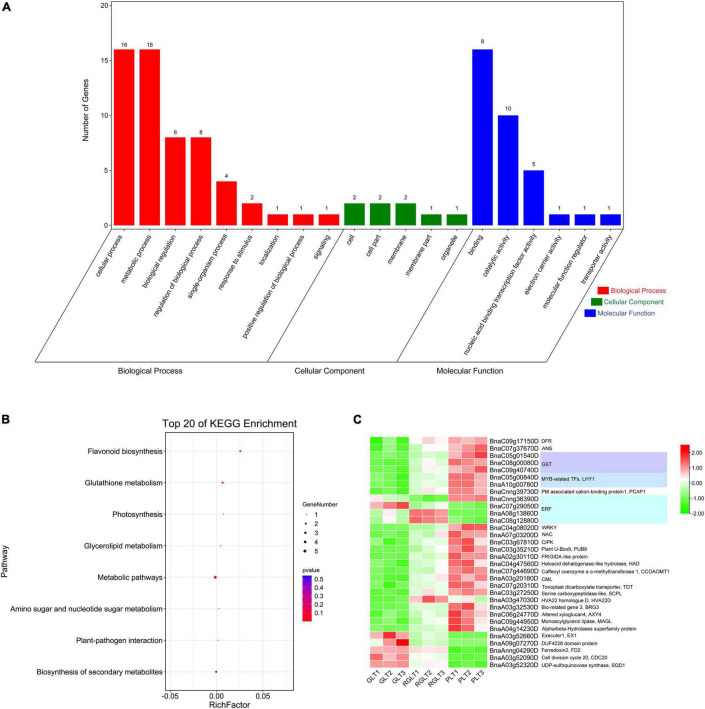
GO and KEGG analysis and gene expression heatmap of the 32 overlapping DEGs in the different colored leaves of *B. napus*. **(A)** GO analysis of the 32 overlapping DEGs. **(B)** KEGG enrichment analysis of the 32 overlapping DEGs. **(C)** The gene expression heatmap of the 32 overlapping DEGs.

To further assess the biological functions of DEGs in the three different colored leaves of *B. napus*, GO and KEGG enrichment analysis of the 32 overlapping DEGs were performed. More specifically, the 32 DEGs were mapped to the GO database and classified into three categories: biological process, cellular component, and molecular function. In the molecular function and biological process categories, 78.13% (25 DEGs) and 56.25% (18 DEGs) of DEGs were enriched, respectively. In the molecular function category, DEGs were enriched in binding (16/25, 64.00%) and catalytic activity (10/24, 40.00%) terms. In the biological process category, approximately 88.89% of DEGs were mapped to metabolic and cellular processes. Only three DEGs were enriched in cellular processes (Figure 5A). Among all DEGs, the proportion involved in GO terms was similar to that of the 32 overlapping DEGs (Supplementary Figure 3A). The KEGG annotation of the overlapping DEGs showed a similar result (Supplementary Figure 3B).

The KEGG annotation showed that the 32 overlapping DEGs of the three groups were enriched in eight KEGG pathways, of which the five genes with 71.34% enrichment included *BnaC07g37670D* (*ANS*), *BnaC09g17150D* (*DFR*), *BnaC09g40740D* (*GST*), *BnaC05g01540D* (*GST*), and *BnaA03g52320D* (*UDP-sulfoquinovose synthase*, *SQD1*) (Figures 5B,C and Supplementary Table 6). The *BnaC07g37670D* (*ANS*) and *BnaC09g17150D* (*DFR*) genes were enriched in the biosynthesis of flavonoid and secondary metabolites. Two *GSTs* (*BnaC09g40740D* and *BnaC05g01540D*) were enriched in glutathione metabolism, *BnaA03g52320D* (*SQD1*) was enriched in glycerolipid metabolism, as well as amino sugar and nucleotide sugar metabolism, *BnaAnng04290D* (*ferredoxin, FD2*) and *BnaA03g20180D* (*calcium-binding protein CML, CML5*) were enriched in photosynthesis and plant-pathogen interaction (Figures 5B,C and Supplementary Table 6). The KEGG annotation of the total DEGs among the GLT vs. PLT, GLT vs. RGLT, and RGLT vs. PLT showed similar results (Supplementary Figure 3B). DEGs in the three groups were primarily enriched in metabolic pathways (147–455 DEGs, 53.40–55.68%), biosynthesis of secondary metabolites (91–259 DEGs, 31.21–36.05%), and flavonoid biosynthesis (4 –13 DEGs, 1.52–3.40%; Supplementary Figure 3B). KEGG annotation showed that the most enrichment pathway were metabolic pathways (i.e., biosynthesis of secondary metabolites, flavonoid biosynthesis, etc.), which might contribute to the formation of the *B. napus* leaf colors.

### Identification of Transcription Factors Related to Anthocyanin Biosynthesis

**FIGURE 6:**
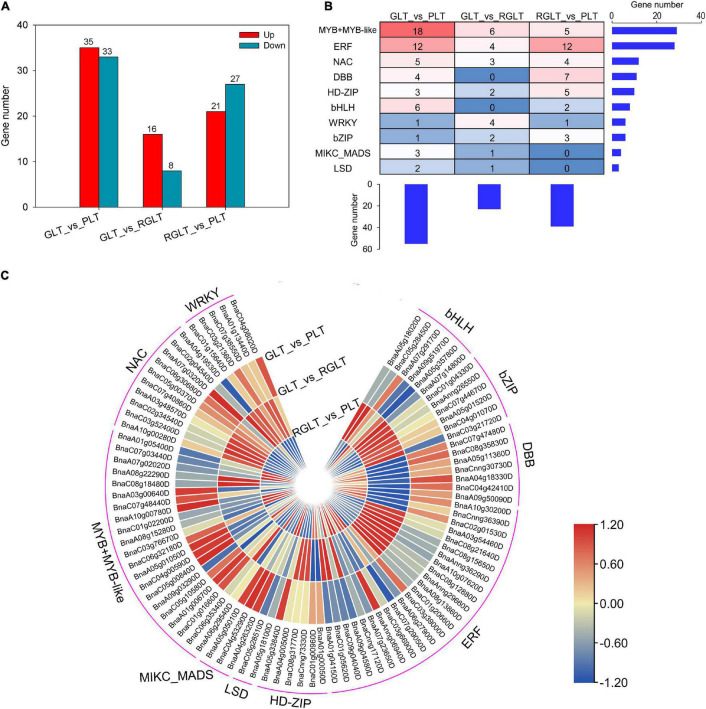
Distinct expression patterns of transcription factors (TFs). **(A)** Number of differentially expressed TFs in the GLT, RGLT, and PLT groups. **(B)** Overview of the enriched TF family. The histograms show the number of genes belonging to each TF family or in each *B. napus* variety. **(C)** Heatmaps represent the expression fold change (Log_2_FC) of TFs among GLT, RGLT, and PLT groups.

To better understand the regulatory networks involved in anthocyanin biosynthesis, differentially expressed TFs were identified. A total of 5,986 TFs in RNA-seq were identified by BLAST analysis of the *B. napus* genome sequence. We found that 68, 24, and 48 TFs were differentially expressed in the GLT vs. PLT, GLT vs. RGLT, and RGLT vs. PLT groups, respectively (Figure 6A). Ten major TF families modulated the gene expression levels among the three *B. napus* varieties (Figures 6B,C). The most TFs were found in the GLT vs. PLT group, while the fewest were observed in the GLT vs. RGLT group, similar to the DEG results among the three varieties (Figure 4A). Among the detected TFs, the members of MYB, ERF, NAC, and bHLH families were more involved in regulating gene transcription (Figures 6B,C). In the GLT vs. PLT, GLT vs. RGLT, and RGLT vs. PLT groups, Log_2_(FC) values of *BnaC05g00840D*, *BnaA10g00780D* (*MYB-related TFs*), *BnaCnng36390D* (*ERF*), *BnaC04g08020D* (*WRKY*), and *BnaA07g03200D* (*NAC*) were similar to those presented in the heatmap (Figure 5). Furthermore, the genes *BnaC06g32180D* (*MYB-like 2*, *MYBL2*), *BnaC01g01660D* (*MYB73*), *BnaA07g29170D* (*bHLH*), *BnaA05g01050D* (*circadian clock associated 1*, *CCA1*) and *BnaC06g30680D* (*NAC*) were strongly upregulated in the GLT vs. PLT, GLT vs. RGLT, and RGLT vs. PLT groups. *BnaAnng06940D* (*ERF104*) and *BnaA10g30200D* (*ERF106*) were significantly upregulated in GLT vs. PLT and RGLT vs. PLT, whereas exhibiting no obvious difference in the GLT vs. RGLT group (Figure 6C). These differentially expressed TFs may play key roles in regulating the structural genes involved in the leaf coloration of *B. napus*.

### Key Differentially Expressed Genes Responsible for the Anthocyanin Biosynthesis Pathway

**FIGURE 7:**
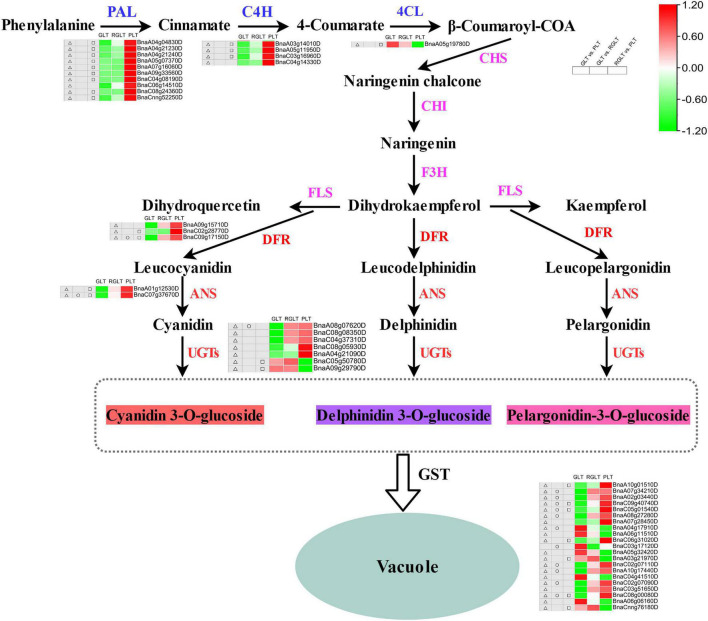
Candidate structural genes related to the anthocyanidin biosynthesis pathway. Heatmap shows the expression levels of GLT, RGLT, and PLT from left to right, respectively. The color change of the heatmap from green to red indicates the expression levels ranging from low to high. △, □, and ○ indicate the differential expressed levels in GLT vs. PLT, GLT vs. RGLT, and RGLT vs. PLT groups, respectively.

To explore the differences in anthocyanin accumulation among the leaves of the three *B. napus* cultivars, DEGs involved in anthocyanin biosynthesis and transport pathways were identified. A total of 27 DEGs were enriched in the anthocyanin synthesis pathway, including *BnPAL*, *Bn4CL*, *BnC4H*, *BnDFR*, *BnANS*, and *BnUGTs*, and 20 *GSTs* were differentially expressed in GLT vs. PLT, GLT vs. RGLT, or RGLT vs. PLT group comparisons (Figure 7). The transcriptional levels of *PAL*, *C4H*, *DFR*, *ANS*, and *UGTs* were significantly upregulated, whereas those of *4CL* and two *UGT* members (*BnaC05g50780D* and *BnaA09g29790D*) were significantly downregulated in the GLT vs. PLT and RGLT vs. PLT groups. The expression levels of *DFR* (*BnaC09g17150D*), *ANS* (*BnaC07g37670D*), and *UGT75C1* (*BnaA08g07620D*) were higher in RGLT than in GLT (Figure 7). Twelve *GSTs* were significantly upregulated, whereas eight were significantly downregulated in the GLT vs. PLT, GLT vs. RGLT, or RGLT vs. PLT groups. Three *GSTs* (*BnaC09g40740D*, *BnaC05g01540D*, and *BnaC08g00080D*) were significantly upregulated in RGLT and PLT groups, compared with the GLT group (Figure 7). Hence, these DEGs may play important roles in the purple leaf formation in *B. napus*, including the regulation of anthocyanin biosynthesis and transport.

To further confirm the DEGs involved in the anthocyanin biosynthesis pathway in *B. napus* leaf, correlation analysis was performed for the DEGs and DAMs in three different colored leaf cultivars. For the correlation analysis, genes with coefficients of |*r*| ≥ 0.8 were selected. A total of 4,187 DEGs were associated with 39 metabolites, of which 1,578–1,598 (37.69–38.17%) were significantly associated with naringenin-7-O-glucoside, pelargonidin-3-O-sophoroside-5-O-(malonyl)-glucoside, petunidin-3-O-(coumaryl)-glucoside, pelargonidin-3-(6-caffeyl sophoroside)-5-glucoside, and peonidin-3-(caffeoyl-glucosyl-glucoside)-5-glucoside (Supplementary Table 7). A total of 1,327 DEGs correlated with all five metabolites, indicating that these metabolites have a similar accumulation tendency (Supplementary Figure 4).

**FIGURE 8:**
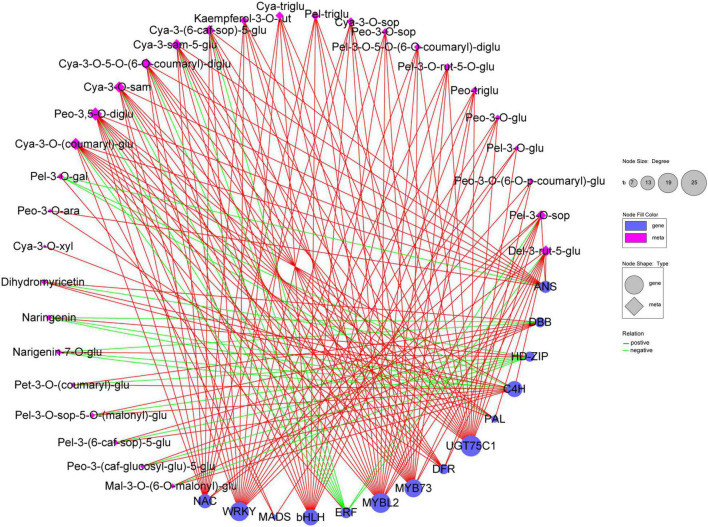
Correlation network of 30 metabolites and 14 key genes involved in flavonoid and anthocyanin biosynthesis in *B. napus* leaves. Blue circle and pink diamond represent genes and metabolites, respectively. The degree represents the number of genes or metabolites. Relation represents the correlations with the coefficient value ≥ 0.80 (positive) or ≤ –0.80 (negative). Cya-3-O-xyl, cyanidin-3-O-xyloside; Cya-3-O-(coumaryl)-glu, cyanidin-3-O-(coumaryl)-glucoside; Cya-3-O-5-O-(6-O-coumaryl)-diglu, cyanidin-3-O-5-O-(6-O-coumaryl)-diglucoside; Cya-3-O-sam, cyanidin-3-O-sambubioside; Cya-3-sam-5-glu, cyanidin-3-O-sambubioside-5-O-glucoside; Cya-3-(6-caf-sop)-5-glu, cyanidin-3-(6-caffeylsophoroside)-5-glucoside; Cya-3-O-xyl, cyanidin-3-O-xyloside; Cya-3-O-sop, Cyanidin-3-O-sophoroside; Cya-triglu, cyanidin-3,5,3’-O-triglucoside; Del-3-rut-5-glu, delphinidin-3-rutinoside-5-glucoside; Kaempferol-3-O-rut, kaempferol-3-O-rutinoside; Mal-3-O-(6-O-malonyl)-glu, malvidin-3-O-(6-O-malonyl-beta-D-glucoside); Pel-3-O-sop-5-O-(malonyl)-glu, pelargonidin-3-O-sophoroside-5-O-(malonyl)-glucoside; Pel-3-O-gal, pelargonidin-3-O-galactoside; Pel-3-(6-caf-sop)-5-glu, pelargonidin-3-(6-caffeylsophoroside)-5-glucoside; Pel-3-O-sop, pelargonidin-3-O-sophoroside; Pel-3-O-5-O-(6-O-coumaryl)-diglu, pelargonidin-3-O-5-O-(6-O-coumaryl)-diglucoside; Pel-triglu, pelargonidin-3,5,3’-O-triglucoside; Pel-3-O-rut-5-O-glu, pelargonidin-3-rutinoside-5-glucoside; Peo-3,5-O-diglu, peonidin-3,5-O-diglucoside; Peo-3-O-ara, peonidin-3-O-arabinoside; Peo-3-(caf-glucosyl-glu)-5-glu, peonidin-3-(caffeoyl-glucosyl-glucoside)-5-glucoside; Pet-3-O-(coumaryl)-glu, petunidin-3-O-(coumaryl)-glucoside; Naringenin-7-O-glu, naringenin-7-O-glucoside; Peo-3-O-sop, peonidin-3-O-sophoroside; Peo-triglu, peonidin-3,5,3’-O-triglucoside; Peo-3-O-glu, peonidin-3-O-glucoside; Pel-3-O-glu, pelargonidin-3-O-glucoside; Peo-3-O-(6-O-p-coumaryl)-glu, peonidin-3-O-(6-O-p-coumaryl)-glucoside.

The correlation network was then used to select the regulatory correlation between metabolites and genes involved in flavonoid and anthocyanin biosynthesis pathways (Figure 8). This result indicated that DEGs were strongly correlated with metabolites. MYB, WRKY, bHLH TFs, and key structural genes (*DFR* and *UGT75C1*) were significantly and positively correlated with metabolites. Moreover, certain TFs (ERF, HD-ZIP) and structural genes (*PAL*, *C4H*, *ANS*) were significantly and negatively correlated with naringenin, naringenin-7-O-glucoside, or pelargonidin-3-O-galactoside. This result demonstrated that these TFs and structural genes may play important roles in flavonoid and anthocyanin biosynthesis.

### Gene Expression Analysis of RNA-Seq by qRT-PCR

**FIGURE 9:**
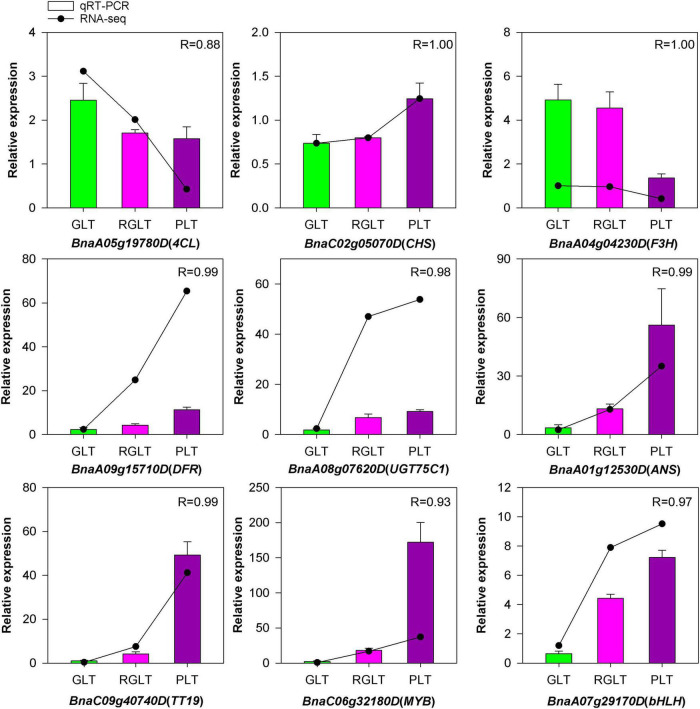
The qRT-PCR analysis of the expression patterns for genes involved in *B. napus* anthocyanin biosynthesis. Green, red, and purple represent the expression levels of genes in GLT, RGLT, and PLT, respectively.

To validate the credibility of the RNA-seq data, we subjected nine DEGs to qRT-PCR. Most of these genes were highly expressed in PLT, while only two genes were highly expressed in GLT or RGLT (Figure 9). Among them, the expression of *BnaC02g05070D* (*CHS*), *BnaA09g15710D* (*DFR*), *BnaA08g07620D* (*UGT75C1*), *BnaA01g12530D* (*ANS*), and *BnaC09g40740D* (*TT19*) were markedly up-regulated in PLT. Similarly, the expression of two TFs (*BnaC06g32180D* and *BnaA07g29170D*), which are the member of MYB and bHLH families, respectively, was markedly enhanced in PLT. In contrast, *BnaA05g19780D* (*4CL*) and *BnaA04g04230D* (*F3H*), which are involved in the phenylpropanoid pathway or the early flavonoid biosynthetic stages, were significantly down-regulated in PLT. Correlation analysis of the expression levels of these genes was significantly correlated with the results of RNA-seq (R > 0.88), validating the sequencing results. Taken together, these results indicate that RNA-seq data accurately assessed and identified genes involved in anthocyanin biosynthesis in *B. napus*.

## Discussion

### Anthocyanin Identification in the Purple Leaves of *Brassica napus*

Anthocyanins, the key metabolites for determining the coloration of fruits, leaves, or petals, can promote health in humans and protect plants against environmental stress (Jaakola, 2013). Breeding functional food and feeding crops rich in anthocyanins has been an ongoing global objective (Asem et al., 2015). In our previous study, we obtained three different colored leaf *B. napus* varieties from green to purple, the coloration of which was primarily caused by anthocyanins (Li et al., 2016). The anthocyanin content in *B. napus* leaves was determined and found to correspond with color intensity, which was 7.66–65.34 mg 100 g^–1^FW in the green to purple leaves of *B. napus* (Figures 1A–C). Furthermore, anthocyanin was accumulated on the adaxial surface of *B. napus* leaves (Figures 1A,B), which is consistent with results of a previous study on *B. napus* (He et al., 2021b), however, inconsistent with those related to *B. juncea* (Heng et al., 2020b) and *B. rapa* (Song et al., 2020b). The marked difference in anthocyanin accumulation and distribution may be due to differences in cultivars and heredity (He et al., 2021b).

Metabolome and transcriptome sequencing have been used as powerful tools to study the molecular mechanisms underlying different biological processes (Wang et al., 2020). In this study, transcriptome and anthocyanins metabolome analysis were used to elucidate anthocyanin biosynthesis in green to purple leaves of *B. napus*. A total of 4 flavonoids and 35 anthocyanins were identified in *B. napus* leaves using the API 6500 QTRAP UPLC/MS/MS system (Figure 2 and Supplementary Table 2). The 35 anthocyanins comprised 10 cyanidins, 8 pelargonidins, 7 peonidins, 4 delphinidins, 4 petunidins, and 2 malvidins. It is well-known that cyanidin, delphinidin, pelargonidin, peonidin, petunidin, and malvidin are common anthocyanin pigments (Jaakola, 2013). In general, cyanidin and peonidin are classified as red pigments, delphinidin, petunidin, and malvidin are categorized as blue pigments (Song et al., 2020b), and pelargonidin as an orange and red pigment (Khoo et al., 2017). Purple color is produced by combining red and blue pigments (Song et al., 2020b). In the present study, six anthocyanin aglycones were detected, however, the three *B. napus* varieties presented different compositions and ratios of anthocyanins. The main forms of anthocyanins were cyanidin and petunidin in PLT and RGLT, whereas delphinidin and petunidin predominated in GLT (Figure 2 and Supplementary Table 2). These results indicate that cyanidin may be main component of purple leaves in *B. napus*, which is consistent with the results of Zhang and Jing (2020), who found that the major anthocyanins in *Brassica* vegetables are derivatives of cyanidin 3-diglucoside-5-glucoside.

Differences in tissue color are typically caused by different anthocyanin types and contents, with higher anthocyanin content in tissues often resulting in a darker shade (Fu et al., 2021). In this study, 22 DAMs among GLT vs. PLT, GLT vs. RGLT, and RGLT vs. PLT were identified according to anthocyanin content, indicating that these 22 anthocyanins contribute to different leaf colors in *B. napus*. Venn diagram analysis and relative anthocyanin content showed that cyanidin-3-O-sambubioside-5-O-glucoside, cyanidin-3-(6-caffeylsophoroside)-5-glucoside, cyanidin-3,5,3’-O-triglucoside, and cyanidin-3-O-(coumaryl)-glucoside were shared among all three groups, in which cyanidin-3,5,3’-O-triglucoside was not detected in GLT; its content was 2.03-fold higher in RGLT vs. PLT group, and was higher than that of other anthocyanins in PLT (Figures 3A,B). This result indicated that cyanidin-3,5,3’-O-triglucoside could be the main form of anthocyanin in PLT, supporting the results of Zhang and Jing (2020). Moreover, malvidin-3-O-(6-O-malonyl-beta-D-glucoside), petunidin-3-O-(coumaryl)-glucoside, pelargonidin-3-(6-caffeylsophoroside)-5-glucoside, pelargonidin-3-O-sophoroside-5-O-(malonyl)-glucoside, and peonidin-3-(caffeoyl-glucosyl-glucoside)-5-glucoside were absent in RGLT and GLT (Figures 3A,B), suggesting that these anthocyanins play important roles in deepening the color of *B. napus* leaves.

Petunidin, delphinidin, pelargonidin, cyanidin, peonidin, and malvidin were present in GLT. The contents of petunidin-3-O-galactoside and delphinidin-3-O-glucoside were nearly equal in the GLT, RGLT, and PLT groups (Figure 2 and Supplementary Table 2), which is consistent with the results of *Salvia miltiorrhiza* flowers reported by Jiang et al. (2020) and turnip skins reported by Zhuang et al. (2019). However, the pigments detected in purple flowers of alfalfa were absent in the cream flowers (Duan et al., 2020). In plants, color is based on the number of pigments, metal ions, or different molecular conformations of anthocyanins (Jiang et al., 2020). These pigments are typically present in small quantities in the green leaves of *B. napus*, thereby indicating that structural genes of anthocyanin metabolic pathways may be expressed at the low levels in the green leaves of *B. napus*.

### Key Structural Genes of Anthocyanin Biosynthesis in Leaves of *Brassica napus*

Many structural and regulatory genes participate in anthocyanin biosynthesis in plants. A single dominant gene controls purple leaf formation in *B. napus* (Li et al., 2016) and *B. juncea* (Heng et al., 2020a). In *B. napus*, the purple leaf formation is controlled by a candidate incomplete dominant gene *BnAPR2*, which encodes adenosine 5′-phosphosulfate reductase (Li et al., 2016). In *B. oleracea*, the purple leaf trait is controlled by a single dominant gene *BoPr*, which is homologous of *DFR* gene in *Arabidopsis* (Liu et al., 2017). These findings suggest that the purple leaf gene involved in the molecular mechanism of anthocyanin biosynthesis may be different in plants, even in closely related species.

Since the discovery of the anthocyanin biosynthesis pathway by Holton and Cornish (1995), the functions of the key structural genes involved in anthocyanin biosynthesis have been explored in plants. Previous studies have reported that early biosynthesis genes (*CHS*, *CHI*, and *F3H*) and late biosynthesis genes (*F3′H*, *F3′5′H*, *DFR*, *ANS*, and *UFGT*) are required for anthocyanin biosynthesis (Goswami et al., 2018; Liu et al., 2018). Furthermore, the late biosynthesis genes and anthocyanin content have been consistently observed in many vegetables (Liu et al., 2018). However, in late biosynthesis genes, *FLS1*, *DFR*, *ANS*, and *UGT75C1* are putative candidates responsible for anthocyanin accumulation in high-anthocyanin resynthesized *B. napus* (Goswami et al., 2018). In this study, 32 DEGs overlapped among the GLT vs. PLT, GLT vs. RGLT, and RGLT vs. PLT comparisons, including *ANS* (*BnaC07g37670D*), *DFR* (*BnaC09g17150D*), and three *GSTs* (*BnaC05g01540D*, *BnaC08g00080D*, and *BnaC09g40740D*) (Figures 5B,C and Supplementary Table 6). The expression levels of most *PAL*, *C4H*, *DFR*, *ANS*, *UGTs* and *GSTs* were significantly upregulated in the GLT vs. PLT and RGLT vs. PLT groups, in which the expression levels of *DFR* (*BnaC09g17150D*) and *ANS* (*BnaC07g37670D*) in PLT were higher than those in GLT and RGLT (Figure 7). Correlation analysis for the DEGs and DAMs, further revealed that the structural genes *DFR* (*BnaC09g17150D*) and *UGT75C1* (*BnaA08g07620D*) were significantly and positively correlated with anthocyanin metabolites, whereas *ANS* (*BnaC07g37670D*) was positively correlated with most anthocyanins, except pelargonidin-3-O-galactoside (Figure 8). *DFR* and *ANS* were significantly upregulated in RGLT and PLT, which might have resulted in anthocyanin accumulation in the purple leaves of *B. napus*. DFR catalyzes the biosynthesis of leucoanthocyanidin, and ANS catalyzes the conversion of leucoanthocyanidins to colored anthocyanidins (Wang et al., 2018). The lack of DFR and ANS activities can lead to anthocyanin accumulation in tobacco (Jiao et al., 2020). UGT75C1 belongs to the phylogenetic group of anthocyanin 5-O-glucosyltransferases and is non-redundant in *Arabidopsis*; its mutant (*ugt75c1*) does not accumulate anthocyanin 5-O-glucosides (Tohge et al., 2005; Si et al., 2022). This result was consistent with the finding that *UGT75C1* is important for anthocyanin biosynthesis in *Acer truncatum*, as reported by Si et al. (2022). Therefore, we speculated that *UGT75C1*, along with other structural genes of anthocyanin biosynthesis, plays an important role in purple leaf formation in *B. napus*. Interestingly, the expression of *4CL* and *F3H* was clearly down-regulated in PLT (Figure 9). In addition, to catalyze p-coumaric acid to form 4-coumaroyl-CoA, 4CL plays an important role in lignin biosynthesis pathway (Jiang et al., 2019). The lower transcript level of *4CL* in PLT may be caused by the decrease of lignin biosynthesis. F3H is one of the nuclear enzymes, catalyzing naringenin to dihydroflavonols. It has been reported that the high expression of *F3H* is associated with the accumulation of flavonols in safflower, whereas its low expression does not affect the increased accumulation of flavonols (Tu et al., 2016). It may imply that other *F3H* genes or transposable elements may positively regulate the accumulation of flavonols and anthocyanins in PLT.

Anthocyanin accumulation in vacuoles is related to anthocyanin transport (Zhao, 2015; Mattioli et al., 2020). In this study, 20 *GSTs* were detected in DEGs of three different colored leaves of *B. napus* cultivars, of which three *GSTs* (*BnaC09g40740D*, *BnaC05g01540D*, and *BnaC08g00080D*) were significantly upregulated in GLT vs. PLT, GLT vs. RGLT, and RGLT vs. PLT (Figure 7). *BnaC09g40740D* encodes an anthocyanin transporter protein, the homologue of *TT19* is significantly and positively correlated with the expression of structural genes in *B. napus* (Goswami et al., 2018). Other homologous genes of *TT19* (*BnaA02g03440D*, *BnaC02g07110D*, and *BnaA10g17440D*) were significantly expressed in GLT vs. PLT and GLT vs. RGLT groups (Figure 7). Therefore, we speculate that the differential expression of *DFR*, *ANS*, and *TT19* contributes to leaf color diversity in *B. napus*.

### Transcription Factors Related to Anthocyanin Biosynthesis

In addition to the key structural genes, flavonoid and anthocyanin biosynthesis is regulated by TFs such as MYB, bHLH, WD40, and MADS-box (Jaakola, 2013). In plants, MYB members are the key TFs regulating anthocyanin biosynthesis. In *Arabidopsis*, MYB75 controls anthocyanin biosynthesis as a master regulator (Solfanelli et al., 2006). MYB111 is a positive regulator of flavonoid biosynthesis, binding to specific cis-elements in the promoters of *CHS*, *F3H*, and *FLS* (Li et al., 2019). In *B. juncea*, the R2R3-MYB TF (*BjPur*) controls the purple leaf formation, and it has been established that a 1,268-bp insertion in the first intron of the *BjPur* gene substantially reduces the expression level of *BjPur* in the green leaves of *B. juncea* (Heng et al., 2020a). In this study, ten, two, and four MYB or MYB-like TFs were significantly regulated in GLT vs. PLT, GLT vs. RGLT, and RGLT vs. PLT, respectively, especially *BnaC05g00840D*, *BnaA10g00780D* (*MYB-related TFs*), the homologous of *late elongated hypocotyl 1* (*LHY1*) in *Arabidopsis*, and *BnaA05g01050D* (*CCA1*) (Figure 6C). It is generally known that CCA1 and LHY1 act as master regulators of the central loop of the circadian clock, which comprises three loops, a central loop and two side loops (Ding et al., 2007; Nguyen and Lee, 2016). Furthermore, the expression of the genes involved in anthocyanin biosynthesis, such as *CHS*, *CHI*, and *DFR*, are regulated by a circadian rhythm (Deikman and Hammer, 1995). In *Arabidopsis*, an MYB-related gene (*MYB-like domain*, *MYBD*) which belongs to the CCA1-like group, functions as a positive regulator of anthocyanin biosynthesis (Nguyen and Lee, 2016). In this study, we found that two *LHY1* genes (*BnaC05g00840D*, *BnaA10g00780D*) and *CCA1* (*BnaA05g01050D*) were significantly upregulated in PLT (Figure 6C). According to this correlation, the expression of *BnaC06g32180D* (*MYBL2*) and *BnaC01g01660D* (*MYB73*) exhibited the positive correlation coefficient with anthocyanins metabolites (Figure 7). Nevertheless, MYBL2 functions as a negative regulator of flavonoid biosynthesis (Dubos et al., 2008), and *MYBD* increases anthocyanin accumulation via repression of *MYBL2* expression in *Arabidopsis* (Nguyen and Lee, 2016), while MYB73 interacts with UV-B photoreceptor UVR8 to regulate auxin responses and lateral root development (Yang et al., 2019). We speculated that one or several MYB-related genes may act as regulators of anthocyanin biosynthesis by the circadian clock in purple leaf of *B. napus*. This speculation still requires further studies to validate it.

Furthermore, MYB interacts with bHLH and WD40 to form the ternary complex MBW, to regulate anthocyanin biosynthesis in plants (Xu et al., 2015). However, the MBW complex is not indispensable for anthocyanin biosynthesis in apple (An et al., 2012). In this study, one bHLH gene (*BnaA07g29170D*) was upregulated in the RGLT and PLT groups (Figure 6); any annotated expression of the WD40 gene was not detected among three *B. napus* varieties (Figure 6). Similar results have been reported in turnip (Zhuang et al., 2019) and in a high-anthocyanin resynthesized *B. napus* (Goswami et al., 2018). Therefore, the functions of these MYB-related TFs in the regulation of anthocyanin biosynthesis requires further study.

Additionally, WRKY TFs have been established to play important roles in the transcriptional regulation of anthocyanin biosynthesis. Previous studies have reported that *WRKY75* can regulate anthocyanin accumulation by activating the promotors of *DFR*, *UFGT*, or *MYB* in pear (Cong et al., 2021), whereas *WRKY44*, as the hub gene, has been demonstrated to regulate the accumulation of anthocyanin in kiwifruit (Peng et al., 2020) and eggplant (He et al., 2021a). In the present study, we found that *BnaC04g08020D*, a homologous of *AtWRKY44* in *Arabidopsis*, was differentially expressed among three assessed *B. napus* varieties (Figures 5C, 6C). In *Arabidopsis*, WRKY44 functioning in conjunction with the MBW complex, has been shown to regulate the anthocyanin and proanthocyanin pathway (Lloyd et al., 2017). We accordingly speculated that *BnaC04g08020D* might regulate anthocyanin pathway by interacting with MYB, MBW, or other TFs in *B. napus*. Additionally, ERF members were the most differentially expressed TFs in the three *B. napus* varieties; and that *NAC*, *DBB*, *ZIP*, and *MADS* TFs also showed significantly different levels of expression among these three cultivars (Figure 6). Moreover, the expression of these TFs was found to be closely correlated with the occurrence of anthocyanin metabolites (Figure 8). Consequently, we speculate that these differentially expressed TFs may play roles in anthocyanin biosynthesis or transport in the purple leaves of *B. napus*, and accordingly, further studies should ideally focus on identifying and verifying the key candidate gene(s) or TF(s) controlling the purple leaf formation in *B. napus*. Gene IDs of these candidate genes in *B. napus* cv. Zhongshuang11 (ZS11) genome V2.0 (Sun et al., 2017; Song et al., 2020a) were shown in Supplementary Table 8.

## Conclusion

In this study, metabolome and transcriptome analyses were used to identify key anthocyanins and candidate genes responsible for the formation of purple leaves in *B. napus*. A total of 35 anthocyanins were detected, including ten cyanidins, eight pelargonidins, seven peonidins, four delphinidins, four petunidins, and two malvidins. Cyanidins, especially cyanidin-3,5,3’-O-triglucoside, as well as other anthocyanins, may represent the main components in purple leaves that contribute to the deepening color of *B. napus* leaves. Moreover, two structural genes (*DFR* and *ANS*) in the anthocyanin biosynthesis pathway, three GSTs (the homology of TT19), and differentially expressed TFs (MYB, bHLH, WRKY, and ERF) were identified as candidate regulators contributing to anthocyanin biosynthesis or transport in *B. napus* leaves. The findings of this study will provide valuable information and new insights for further investigations of the regulatory network underlying the accumulation of anthocyanin in *B. napus*.

## Data Availability Statement

The LC-MS data are deposited in the MetaboLights database under accession number MTBLS4945. The RNA-seq data are deposited in the NCBI Sequence Read Archive (SRA) under accession number PRJNA822841.

## Author Contributions

HL and HF contributed to the study conception and design. YD performed the sample collection and RNA isolation. JZ and GY performed the gene expression analysis. YZ and JL performed the bioinformatics analysis. YZ and HL wrote the manuscript. All authors have contributed to the manuscript, read, and approved the submission of the final manuscript.

## Conflict of Interest

The authors declare that the research was conducted in the absence of any commercial or financial relationships that could be construed as a potential conflict of interest.

## Publisher’s Note

All claims expressed in this article are solely those of the authors and do not necessarily represent those of their affiliated organizations, or those of the publisher, the editors and the reviewers. Any product that may be evaluated in this article, or claim that may be made by its manufacturer, is not guaranteed or endorsed by the publisher.

## Footnotes

1
http://www.omicshare.com/tools/Home/Soft/gogsea

## Supplementary Material

Supplementary Figure 1 Log_2_(FC) of DAAs among GLT vs PLT, GLT vs RGLT, and RGLT vs PLT. **(A)** Log_2_(FC) of DAAs in GLT vs PLT. **(B)** Log_2_(FC) of DAAs in GLT vs RGLT. **(C)** Log_2_(FC) of DAAs in RGLT vs PLT.

Supplementary Figure 2 Sample correlation analysis and comparative analysis of DEGs. **(A)** Pearson correlation coefficient among GLT, RGLT, and PLT. **(B)** The numbers of differently up-regulated and down-regulated genes among GLT vs PLT, GLT vs RGLT, and RGLT vs PLT.

Supplementary Figure 3 GO, KEGG analysis, and gene expression heatmap of the total DEGs in different color leaves of *B. napus*. **(A)** GO analysis of the total DEGs. **(B)** KEGG enrichment analysis of the total DEGs.

Supplementary Figure 4 The Venn analysis of DEGs correlation with overlapping 5 DAMs in three cultivars of *B. napus* leaves.

## Abbreviations

DAMs: differentially accumulated metabolites
DEGs: differentially expressed genes
FC: foldchange
TF: transcription factor
PAL: phenylalanine ammonialyase
4CL: 4-coumaryol CoA ligase
C4H: cinnamate 4-hydroxylase
CHS: chalcone synthase
CHI: chalcone isomerase
DFR: dihydroflavonol 4-reductase
F3H: flavanone 3-hydroxylase
F3′H: flavonoid 3′-hydroxylase
F3′5′H: flavonoid 3′,5′-hydroxylase
ANS: anthocyanidin synthase
GST: glutathione S-transferases
GAPDH: glyceraldehyde-3-phosphate dehydrogenase
GT: glucosyltransferases
AT: acyltransferases
MT: methyltransferases
ER: endoplasmic reticulum
bHLH: basic helix–loop–helix
TT8: transparent testa 8
RNA-seq: RNA sequencing
GO: Gene Ontology
MYBD: MYB-like Domain transcription factor
MYBL2: MYB-like 2
CCA1: circadian clock associated 1
LHY1: late elongated hypocotyl 1.
