# Supplementary material for: Unraveling the Mechanism of Purple Leaf Formation in Brassica napus by Integrated Metabolome and Transcriptome Analyses
Source: Front Plant Sci. 2022 Jul 12;13:945553. doi: 10.3389/fpls.2022.945553 (PMC9315442; doi:10.3389/fpls.2022.945553)
Supplement: Supplementary file 1 [file Table_1.DOCX]

**Supplementary Table S1 The corresponding primers of qRT-PCR**

| Gene name | Forward primer | Reverse primer |
| --- | --- | --- |
| BnaA05g19780D | TGCTCACTCTTTCCTCGCTG | GTCGGGTACGCAAACAATCA |
| BnaC02g05070D | AGTCTGTGGAAGATGGCGTAG | AGACAGGAACGCTGTGTAGG |
| BnaA04g04230D | CCCACGTACCCTGTAAGCAA | ACCCATCAGCCTCTCGCTAT |
| BnaA09g15710D | ACAAAGTTCCGGGCAGTGAT | CCATCCCTGGTTCGGTCTTC |
| BnaA08g07620D | TCTTCCGACGATGGTAAAGCC | TTTCTCCCACAAACCGTCCC |
| BnaA01g12530D | AGATCTCTCACTTTGGCCTA | ACTTCATTCTCTAGACGGTCA |
| BnaC09g40740D | TTGAGGTGGGTTACTTCAACGTTTT | ATGTCCAAGACCACCCCGA |
| BnaC06g32180D | TGGACGAACCGACAACGAAG | TGTTGAGGTCAGGCAACCAG |
| BnaA07g29170D | TACCTCACTCTCGGCACTCT | AGGATCTTGCCGCTCCAATC |
| Actin7(BnaA02g00190D) | CTTCCTCACGCTATCCTCCG | AGCCGTCTCCAGCTCTTGC |
| GAPDH(BnaC03g33610D) | CAGGTTTGGAATTGTCGAGG | GAGCTGTGGAAGCACCTTTC |
