# Supplementary material for: Unraveling the Mechanism of Purple Leaf Formation in Brassica napus by Integrated Metabolome and Transcriptome Analyses
Source: Front Plant Sci. 2022 Jul 12;13:945553. doi: 10.3389/fpls.2022.945553 (PMC9315442; doi:10.3389/fpls.2022.945553)
Supplement: Supplementary file 3 [file Table_3.DOCX]

**Supplementary Table S3 Quality inspection of sample sequencing data.**

| **Sample** | **Total reads (bp)** | **Total bases (bp)** | **Clean reads (bp)** | **Clean bases (bp)** | **Q20 rate**  **(%)** | **Q30 rate (%)** | **GC (%)** |
| --- | --- | --- | --- | --- | --- | --- | --- |
| GLT1 | 47,880,324 | 7,182,048,600 | 47,880,324 | 7,176,951,056 | 97.14 | 92.34 | 47.46 |
| GLT2 | 47,766,494 | 7,164,974,100 | 47,766,494 | 7,159,500,634 | 97.12 | 92.26 | 46.91 |
| GLT3 | 46,772,140 | 7,015,821,000 | 46,772,140 | 7,010,782,042 | 96.86 | 91.65 | 47.48 |
| RGLT1 | 46,576,616 | 6,986,492,400 | 46,576,616 | 6,981,436,692 | 97.03 | 92.04 | 47.50 |
| RGLT2 | 46,556,414 | 6,983,462,100 | 46,556,414 | 6,978,160,754 | 97.06 | 92.12 | 47.37 |
| RGLT3 | 46,961,506 | 7,044,225,900 | 46,961,506 | 7,039,015,442 | 97.07 | 92.14 | 46.49 |
| PLT1 | 47,786,112 | 7,167,916,800 | 47,786,112 | 7,161,605,722 | 97.21 | 92.51 | 47.03 |
| PLT2 | 47,558,998 | 7,133,849,700 | 47,558,998 | 7,126,801,908 | 96.97 | 92.08 | 47.69 |
| PLT3 | 46,703,082 | 7,005,462,300 | 46,703,082 | 6,999,970,268 | 97.16 | 92.37 | 47.24 |
