# Supplementary material for: Unraveling the Mechanism of Purple Leaf Formation in Brassica napus by Integrated Metabolome and Transcriptome Analyses
Source: Front Plant Sci. 2022 Jul 12;13:945553. doi: 10.3389/fpls.2022.945553 (PMC9315442; doi:10.3389/fpls.2022.945553)
Supplement: Supplementary file 4 [file Table_4.DOCX]

**Supplementary Table S4 Detection of sample alignment rate.**

| Sample | Total reads | Unmapped  (%) | Uniquely Mapped  (%) | Multiple Mapped  (%) | Total Mapped  (%) |
| --- | --- | --- | --- | --- | --- |
| GLT1 | 47,880,324 | 3,606,172 (7.53%) | 41,629,850 (86.95%) | 2,644,302 (5.52%) | 44,274,152 (92.47%) |
| GLT2 | 47,766,494 | 3,650,834 (7.64%) | 41,169,742 (86.19%) | 2,945,918 (6.17%) | 44,115,660 (92.36%) |
| GLT3 | 46,772,140 | 3,626,261(7.75%) | 40,634,710 (86.88%) | 2,511,169 (5.37%) | 43,145,879 (92.25%) |
| RGLT1 | 46,576,616 | 3,537,768(7.60%) | 40,585,213 (87.14%) | 2,453,635 (5.27%) | 43,038,848 (92.40%) |
| RGLT2 | 46,556,414 | 3,613,449 (7.76%) | 40,506,057 (87.00%) | 2,436,908 (5.23%) | 42,942,965 (92.24%) |
| RGLT3 | 46,961,506 | 3,841,481 (8.18%) | 40,615,747 (86.49%) | 2,504,278 (5.33%) | 43,120,025 (91.82%) |
| PLT1 | 47,786,112 | 3,857,725 (8.07%) | 41,414,954 (86.67%) | 2,513,433 (5.26%) | 43,928,387 (91.93%) |
| PLT2 | 47,558,998 | 3,957,304 (8.32%) | 41,295,375 (86.83%) | 2,306,319 (4.85%) | 43,601,694 (91.68%) |
| PLT3 | 46,703,082 | 3,791,468 (8.12%) | 40,719,446 (87.19%) | 2,192,168 (4.69%) | 42,911,614 (91.88%) |
