# Supplementary material for: Unraveling the Mechanism of Purple Leaf Formation in Brassica napus by Integrated Metabolome and Transcriptome Analyses
Source: Front Plant Sci. 2022 Jul 12;13:945553. doi: 10.3389/fpls.2022.945553 (PMC9315442; doi:10.3389/fpls.2022.945553)
Supplement: Supplementary file 5 [file Table_5.DOCX]

**Supplementary Table S5 Assessment of sample alignment area.**

| **Sample** | **Exon** | **Intron** | **Intergenic** |
| --- | --- | --- | --- |
| GLT1 | 42,644,863 (96.32%) | 296,637 (0.67%) | 1,332,652 (3.01%) |
| GLT2 | 42,408,384 (96.13%) | 339,691 (0.77%) | 1,367,585 (3.10%) |
| GLT3 | 41,450,246 (96.07%) | 302,021 (0.70%) | 1,393,612 (3.23%) |
| RGLT1 | 41,158,050 (95.63%) | 352,919 (0.82%) | 1,527,879 (3.55%) |
| RGLT2 | 41,100,712 (95.71%) | 309,189 (0.72%) | 1,533,064 (3.57%) |
| RGLT3 | 41,166,688 (95.47%) | 319,088 (0.74%) | 1,634,249 (3.79%) |
| PLT1 | 42,342,572 (96.39%) | 311,892 (0.71%) | 1,273,923 (2.90%) |
| PLT2 | 41,892,508 (96.08%) | 331,373 (0.76%) | 1,377,814 (3.16%) |
| PLT3 | 41,044,959 (95.65%) | 386,205 (0.90%) | 1,480,451 (3.45%) |
