# Supplementary figures and images for: Unraveling the Mechanism of Purple Leaf Formation in Brassica napus by Integrated Metabolome and Transcriptome Analyses
Source: Front Plant Sci. 2022 Jul 12;13:945553. doi: 10.3389/fpls.2022.945553 (PMC9315442; doi:10.3389/fpls.2022.945553)

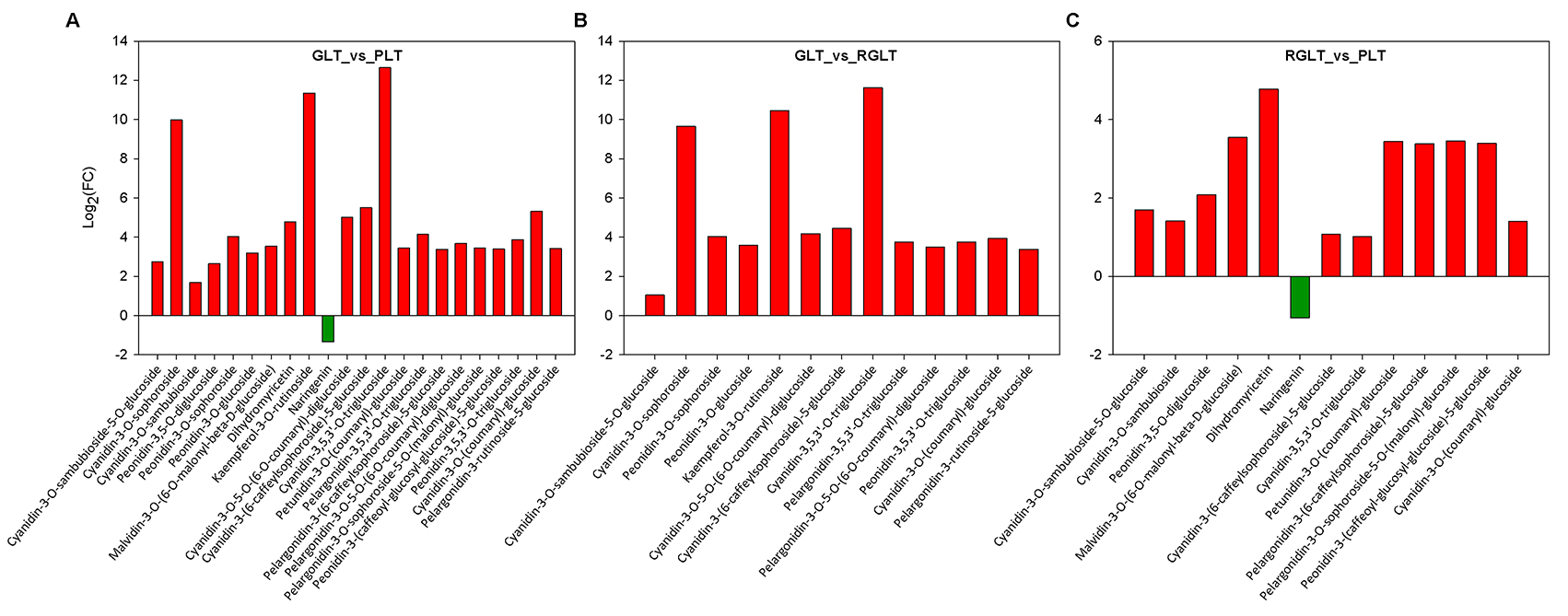

Supplement: Supplementary Figure 1 — Log2(FC) of DAAs among GLT vs PLT, GLT vs RGLT, and RGLT vs PLT. (A) Log2(FC) of DAAs in GLT vs PLT. (B) Log2(FC) of DAAs in GLT vs RGLT. (C) Log2(FC) of DAAs in RGLT vs PLT. [file Image_1.TIF]

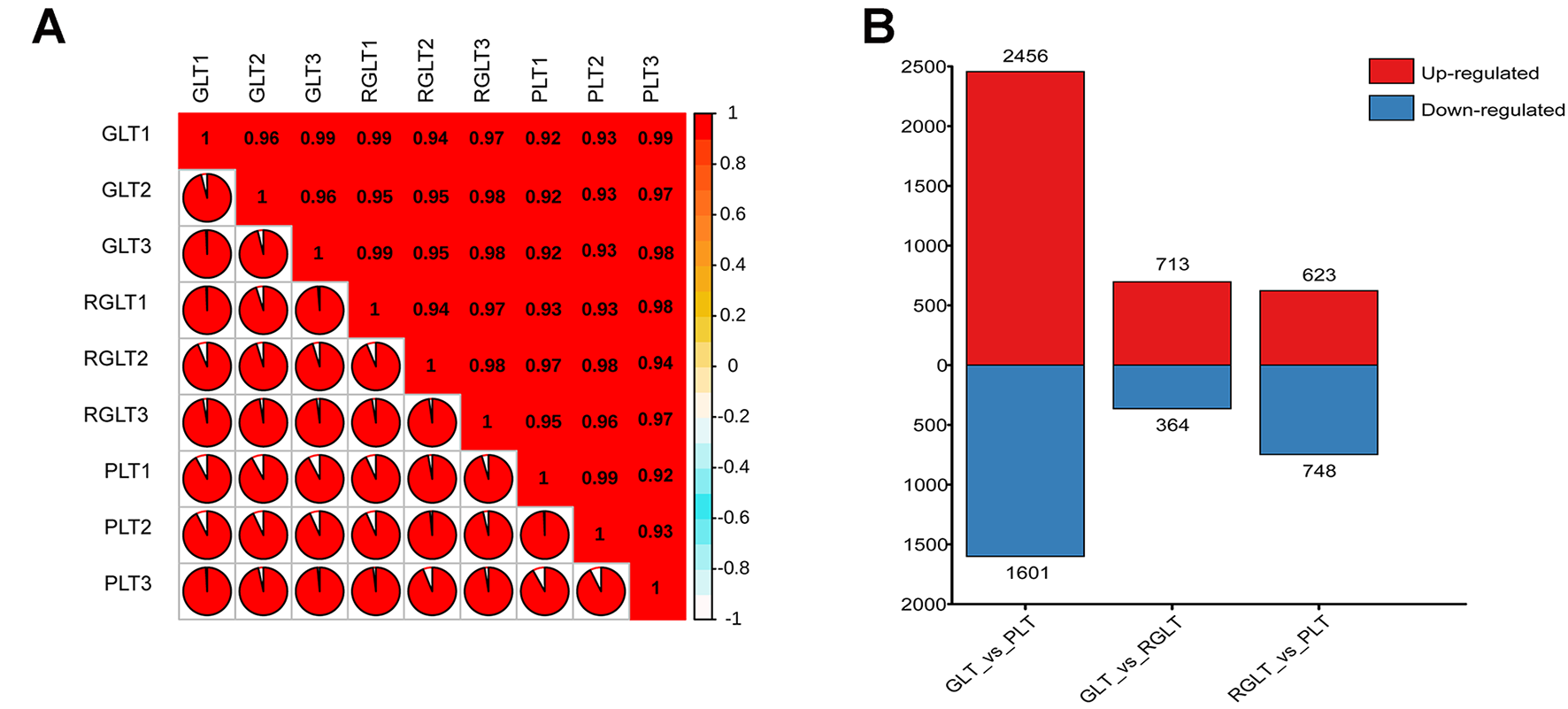

Supplement: Supplementary Figure 2 — Sample correlation analysis and comparative analysis of DEGs. (A) Pearson correlation coefficient among GLT, RGLT, and PLT. (B) The numbers of differently up-regulated and down-regulated genes among GLT vs PLT, GLT vs RGLT, and RGLT vs PLT. [file Image_2.TIF]

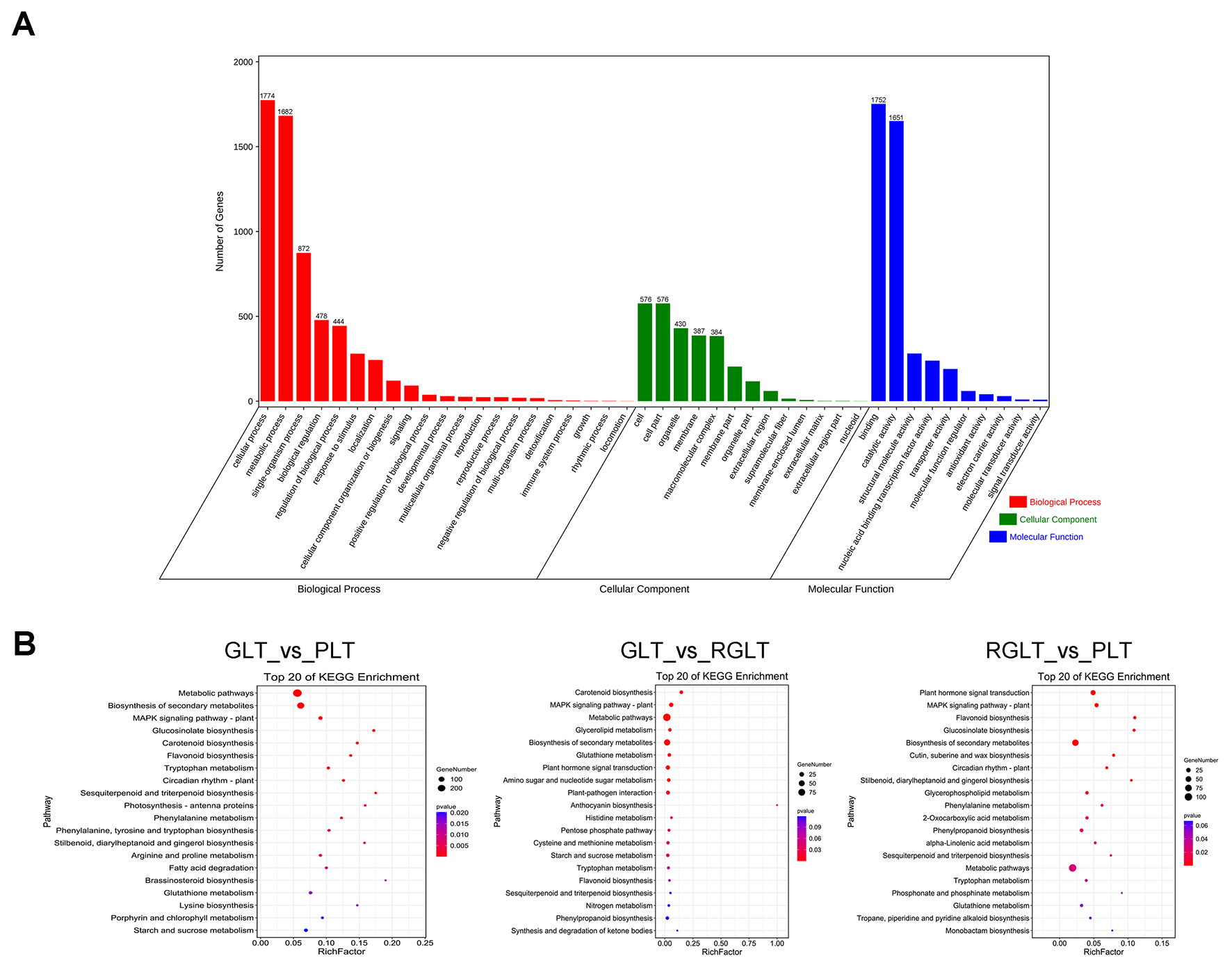

Supplement: Supplementary Figure 3 — GO, KEGG analysis, and gene expression heatmap of the total DEGs in different color leaves of B. napus. (A) GO analysis of the total DEGs. (B) KEGG enrichment analysis of the total DEGs. [file Image_3.TIF]

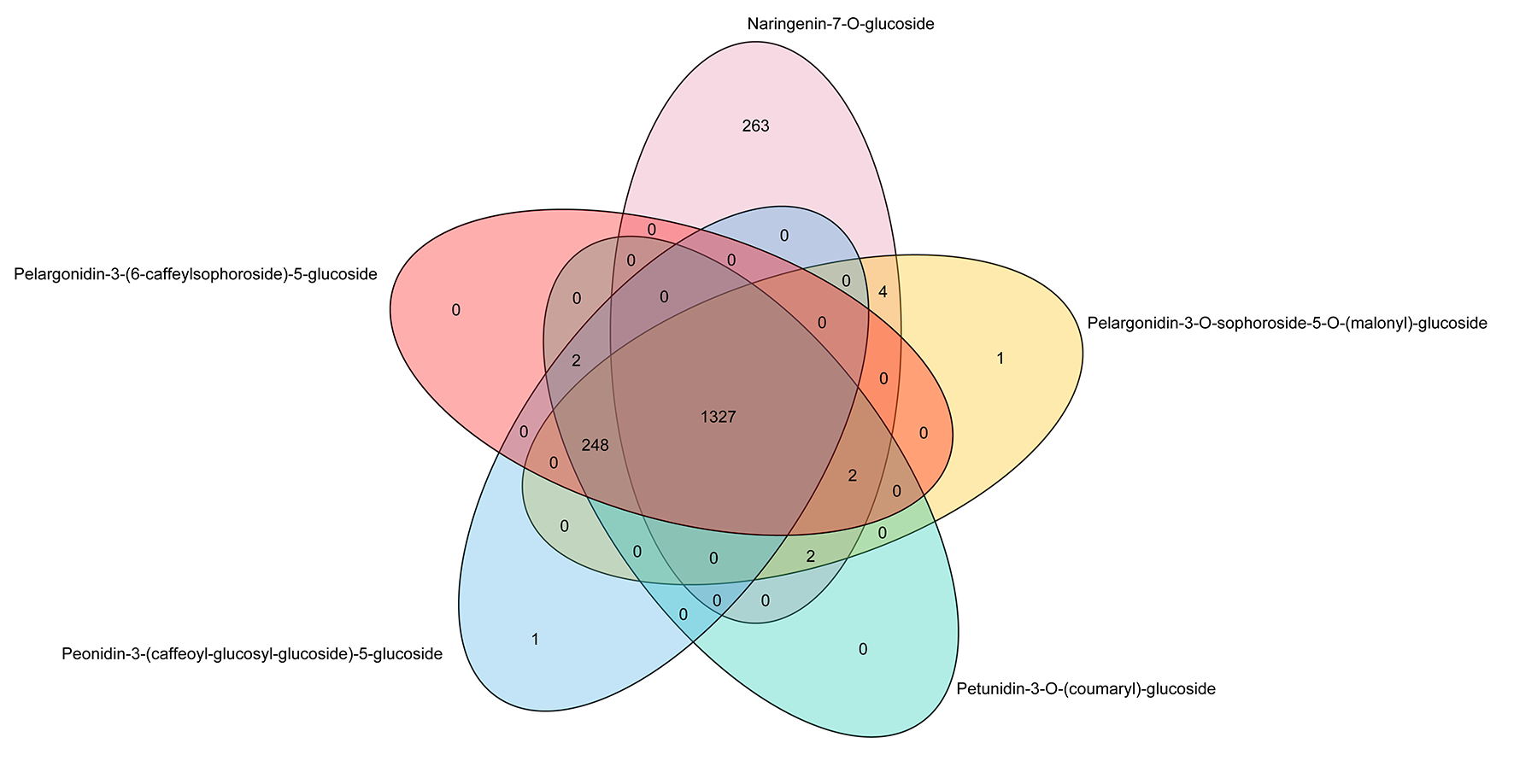

Supplement: Supplementary Figure 4 — The Venn analysis of DEGs correlation with overlapping 5 DAMs in three cultivars of B. napus leaves. [file Image_4.TIF]
